# Ocular complications of plasma cell dyscrasias

**DOI:** 10.1177/11206721231155974

**Published:** 2023-02-09

**Authors:** Rohan Bir Singh, Sachi Singhal, Shruti Sinha, Junsang Cho, Anne Xuan-Lan Nguyen, Lovedeep Singh Dhingra, Snimarjot Kaur, Vasudha Sharma, Aniruddha Agarwal

**Affiliations:** 1 1866 Massachusetts Eye and Ear, Department of Ophthalmology, Harvard Medical School, Boston, MA, USA; 2 Department of Ophthalmology, Great Ormond Street Institute of Child Health, 4919 University College London, London, UK; 3 Discipline of Ophthalmology and Visual Sciences, Faculty of Health and Medical Sciences, University of Adelaide, Adelaide, Australia; 4 Department of Internal Medicine, 22901 Crozer-Chester Medical Center, Upland, PA, USA; 5 Department of Ophthalmology, Vanderbilt Eye Center, Vanderbilt University Medical Center, Nashville, TN, USA; 6 Faculty of Medicine, McGill University, Montreal, Canada; 7 Center for Outcomes Research and Evaluation, 25047 Yale-New Haven Hospital, New Haven, CT, USA; 8 Department of Pediatrics, 25047 Yale-New Haven Hospital, New Haven, CT, USA; 9 Department of Internal Medicine, 29761 Dayanand Medical College and Hospital, Ludhiana, India; 10 Department of Ophthalmology, University of Maastricht, Maastricht, the Netherlands; 11 Department of Ophthalmology, The Eye Institute, Cleveland Clinic Abu Dhabi, Abu Dhabi, United Arab Emirates

**Keywords:** Retinal degenerations associated with systemic disease, RETINA, corneal degenerations, CORNEA / EXTERNAL DISEASE, GLAUCOMA, RETINA, CORNEA / EXTERNAL DISEASE

## Abstract

Plasma cell dyscrasias are a wide range of severe monoclonal gammopathies caused by pre-malignant or malignant plasma cells that over-secrete an abnormal monoclonal antibody. These disorders are associated with various systemic findings, including ophthalmological disorders. A search of PubMed, EMBASE, Scopus and Cochrane databases was performed in March 2021 to examine evidence pertaining to ocular complications in patients diagnosed with plasma cell dyscrasias. This review outlines the ocular complications associated with smoldering multiple myeloma and monoclonal gammopathy of undetermined significance, plasmacytomas, multiple myeloma, Waldenström's macroglobulinemia, systemic amyloidosis, Polyneuropathy, Organomegaly, Endocrinopathy, Monoclonal gammopathy and Skin changes (POEMS) syndrome, and cryoglobulinemia. Although, the pathological mechanisms are not completely elucidated yet, wide-ranging ocular presentations have been identified over the years, evolving both the anterior and posterior segments of the eye. Moreover, the presenting symptoms also help in early diagnosis in asymptomatic patients. Therefore, it is imperative for the treating ophthalmologist and oncologist to maintain a high clinical suspicion for identifying the ophthalmological signs and diagnosing the underlying disease, preventing its progression through efficacious treatment strategies.

## Introduction

Plasma cell dyscrasias, or plasma cell proliferative diseases, are a wide range of severe monoclonal gammopathies caused by pre-malignant or malignant plasma cells that over-secrete an abnormal monoclonal antibody (or a portion of the antibody).^1^ These diseases have a broad clinical spectrum ranging from silent monoclonal gammopathy of undetermined significance (MGUS) to multiple myeloma, Waldenström macroglobulinemia, or other B cell-associated hematological malignancies. The plasma cells derived from B lymphocytes are a critical component of the adaptive immune system and function through the generation of antibodies. These antibodies initiate neutralizing specific antigens typically expressed on the surface of pathogens and foreign substances.

Each clonal plasma cell line is committed to synthesizing a specific immunoglobulin antibody that consists of two identical heavy chains [gamma (γ), mu (μ), alpha (α), delta (δ), or epsilon (σ)] and a light chain [kappa (κ) or lambda (λ)].^2^ Typically, the overproduced light chains are removed from the body as free polyclonal light chains (≤40 mg/24 h) in the urine.^3^ Although, the etiology of plasma cell disorders is not fully elucidated yet, they are characterized by an excessive proliferation of one clone of plasma cells, leading to an increase in the serum level of the clone's product, the monoclonal immunoglobulin protein (M-protein).^4^ M-proteins consist of either one type of chain or both heavy and light chains. Accumulation of M-protein causes several systemic complications, including ocular symptoms.

Paraneoplastic complications can occur in plasma cell dyscrasias regardless of tumor burden, protein levels, or criteria suggesting malignant phase disease.^5^ Plasma cell dyscrasias can progress to the malignant stage characterized by excessive tumor cell burden, causing symptoms and rapid, life-threatening disease findings. Several distinct subsets of malignant dyscrasias exist, including solitary plasmacytoma, non-secretory multiple myeloma, multiple myeloma, light chain multiple myeloma, and plasma cell leukemia.

A search of PubMed, EMBASE, Scopus and Cochrane databases was performed in March 2021 to examine evidence pertaining to ocular complications in patients diagnosed with plasma cell dyscrasias. While wide-ranging systemic involvement of plasma cell dyscrasias has been reported in the literature, there is a lack of information exploring ocular complications. We focus on pre-malignant conditions, including smoldering MM and MGUS, plasmacytomas, multiple myeloma, Waldenström's macroglobulinemia, systemic amyloidosis, POEMS syndrome, and cryoglobulinemia. We outline the ocular complications of plasma cell dyscrasias to assist in identifying and diagnosing, preventing progression, and providing treatment strategies. (Table 1)

**Table 1.** The table summarizing the systemic and ocular presentations observed in patients with plasma cell dyscrasias.

Disorder	Systemic presentation	Ocular manifestations
Monoclonal gammopathy of undetermined significance	Asymptomatic, incidentally diagnosed when being screened for other disorders	Nummular or crystalline opacifications Monoclonal gammopathy of ocular significance Neurosensory macular detachment Retinopathy, retinal detachment, retinal hemorrhages, cottonwool spots, retinal venous dilation Neovascularization of optic disc Vitreous hemorrhage Copper deposition in lens and Descemet's membrane
Smoldering Multiple Myeloma	Asymptomatic, incidentally diagnosed	Ophthalmic vein thrombosis Vortex and crystalline keratopathy
Plasmacytoma	Symptoms related to location of the mass. Majority involve upper respiratory tract causing epistaxis, rhinorrhea, nasal obstruction	Orbit, iris, conjunctival and eyelid plasmacytomas Lacrimal sac involvement
Multiple Myeloma	Anemia, Bone pain, Kidney injury and dysfunction Fatigue and generalized weakness Elevated calcium levels Weight loss	Necrotizing xanthrogranulomas (NXG), Ecchymosis, superficial planar xanthomatosis, necrobiotic xanthogranulomas, necrotizing eyelid lesion Salmon conjunctival patch Light chain deposits in the cornea, vortex keratopathy, paraproteinemic keratopathy Deposition of myelomatous crystals Anterior stromal haze of unknown significance Dense copper accumulation in Descemet membrane Posterior scleritis Bilateral microcysts of the iris Ciliary epithelium cysts Nongranulomatous uveitis Recurrent iridocyclitis Neurosensory macular detachment Pseudovitelliform subretinal deposits Central retinal vein occlusion Retinal hemorrhages, venous congestion, cotton wool spots, microaneurysms Optic nerve infiltration, disc swelling Cranial nerve infiltration (II, III, VI), extraocular muscle infiltration Temporal arteritis, retinal vasculitis
Waldenström's macroglobulinemia	Weakness, Fatigue, Weight loss, Chronic hemorrhage from gums Epistaxis Neurological complaints	Dilatation of retinal vessels, intraretinal hemorrhages, retinal vein occlusions Optic disc edema Neurosensory macular detachment, edema Malignant vitreitis Conjunctival infiltration
Systemic Amyloidosis	Symptoms determined by type of protein and distribution Waxy skin, easy bruising Enlarged muscles Heart failure, conduction abnormalities Hepatomegaly Nephrotic syndrome neuropathies	Glaucoma, Swollen eyelids, conjunctival masses, waxy eyelid papules with purpura, proptosis, diplopia, ptosis, accommodative paresis keratoconjunctivitis sicca, and upper lid masses Ophthalmoplegia Drusenoid deposits Serous retinal detachment Enlargement of choroid vessels, pachychoroid, sub-retinal fluid deposition, bilateral lacrimal deficiency, and occlusion of choriocapillaris Lattice stromal dystrophy
POEMS syndrome	Peripheral polyneuropathy Osteosclerotic lesions Skin changes Organomegaly, endocrinopathies	Optic disc edema Retinal hemorrhage, cotton wool spots, retinal detachment, and macular edema. Central retinal artery occlusion, chalky white optic disc, and choroidal filling defects
Cryoglobulinemia	Purpura, Arthralgia, and weakness associated with cryoglobulinemic vasculitis Skin ulcers, Raynaud's phenomenon, arthritis, sicca syndrome, peripheral neuropathy. Renal involvement −15–30%	Retinal hemorrhages, cotton wool spots Retinal vein occlusion Central retinal artery occlusion Anterior segment ischemia Anterior uveitis Purtscher's retinopathy Necrotizing scleritis Peripheral ulcerative keratitis Subepithelial corneal deposits

## Pre-Multiple Myeloma

### Monoclonal gammopathy of undetermined significance and smoldering multiple myeloma

**Figure 1.**
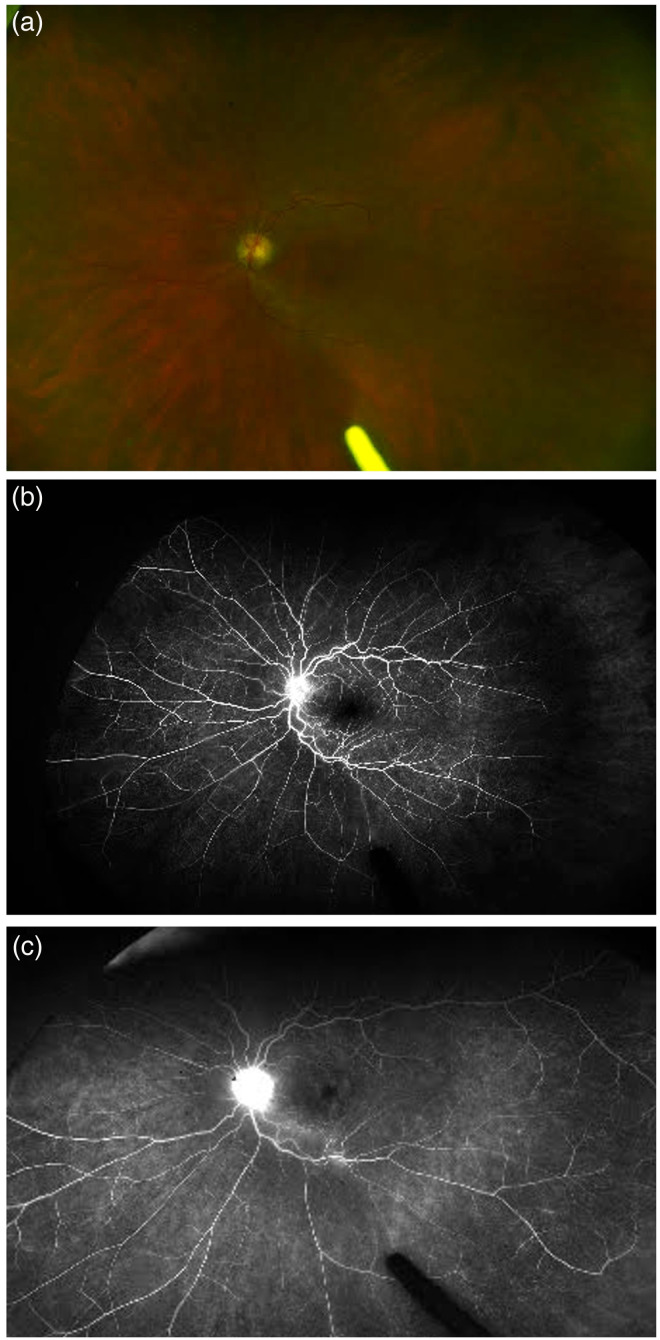
The figure shows fundus photograph and fluorescein angiography (fa) of a 62-year-old female patient with monoclonal gammopathy of undetermined significance (MGUS). There is presence of optic disc hyperemia on fundus photograph (a), with a dull foveal reflex suggestive of central macular edema. The FA shows corresponding leakage in the optic nerve and the macula, along with some leakage from the vessels in the early (b) and late phase (c).

Monoclonal gammopathy of undetermined significance (MGUS) is the presence of monoclonal immunoglobulins (M-protein) in the serum or urine, typically in asymptomatic patients or with signs of other dyscrasias. It is often diagnosed incidentally with serum protein electrophoresis.^6^ In ∼3% of the population over 50, MGUS is the most common type of plasma cell dyscrasia.^6^ While still considered a pre-malignant condition, smoldering multiple myeloma (SMM) is the next stage in the spectrum of conditions. The monoclonal gammopathies are precursors of active multiple myeloma (MM) and typically present as incidental findings on serum protein electrophoresis.^7^ Monoclonal gammopathies, secondary to blood hyperviscosity or direct disease localization, can lead to systemic disease affecting multiple organs, including the eye (Figure 1).

Paraproteinemic keratopathy (PPK) is the corneal involvement in cases of MGUS. It is characterized by distinct corneal crystalline or non-crystalline deposits in various corneal layers. The intracorneal crystalline deposits have been shown to consist of immunoglobulins (predominantly IgG).^8^ Corneal involvement has been reported in a few MGUS cases with diverse presentations.^8–10^ The diverse presentations are attributed to variability in the structure of the light chains and patient-specific factors like corneal endothelial viability.^11,12^ Paraproteinemic keratopathy presents with corneal opacities similar to corneal dystrophies and degenerative corneal disorders. These opacities can be central or peripheral and form patterns such as punctiform, diffuse, lattice-like, granular, or geographic opacities at the epithelial, subepithelial, Descemet's, or stromal layers of the cornea.^9,13^ Anterior segment imaging may be used to identify deposits, and corrective procedures such as penetrating keratoplasty can be performed. However, no interventions are required if opacities are peripheral and involvement is mild.^14^ The term “monoclonal gammopathy of ocular significance” has been proposed for patients with MGUS with ocular findings only. Corneal transplantation may be required if the keratopathy causes binocular visual loss, but the crystalline keratopathy has been reported to recur within one year in the graft.^15^

Retinal involvement in MGUS can manifest as neurosensory macular detachment with angiographically silent and treatment-resistant sub-macular fluid. Patients with a pre-existing chorioretinal disease are more likely to develop it with sub-retinal fluid accumulation.^16–19^ Additionally, autoimmune retinopathy characterized by photoreceptor dysfunction and the presence of anti-retinal autoantibodies has been reported in MGUS. It can present with progressive decline in visual acuity, photopsias, nyctalopia, metamorphopsia, or visual field defects.^20^ Copper deposition in the anterior lens capsule and the Descemet's membrane has also been observed with MGUS rarely, which may be caused by preferential binding of monoclonal antibodies to copper and transferring to thick ocular basement membranes. The exact pathogenic mechanism is unknown, but cataract extraction with anterior capsule removal helps vision restoration.^21,22^

While most of these ocular symptoms can be present throughout the monoclonal gammopathy spectrum, the reported cases of ocular involvement in SMM exclusively are limited. These symptoms might occur due to superior ophthalmic vein thrombosis, vortex, and crystalline keratopathy.^23–26^ Although, corneal involvement is not well recognized in MM, its involvement in MGUS and SMM is well described, with crystalline deposits detected in up to 1% of patients with MGUS.^27,28^ On performing in vivo confocal microscopy (IVCM), the deposits are seen throughout the corneal thickness, showing needle-shaped, round or hexagonal crystalline morphologies. The histopathological examination shows various features of intra- and extracellular tubules, fibrils, and crystals.^27^ Majority of the symptoms in MM are attributed to blood hyperviscosity; however, hyperviscosity retinopathy is rarely reported in MGUS or SMM due to the lower circulating protein concentration.^29^ In the cornea, diffusion from the aqueous humor, tear film, or perilimbal vessels is suspected to be the source of the monoclonal proteins.^27^ Given the risk of MGUS and SMM transforming into overt myeloma, the early recognition of these subtle ocular findings enables prompt diagnosis and management of the malignancy.

### Plasmacytoma

A plasmacytoma is an isolated tumor of bone or soft tissue monoclonal plasma cells without any systemic involvement. It can present either as a solitary lesion or multiple masses in the bone or extramedullary tissue. Despite a mean age of onset in the sixth and seventh decades of life, it has been reported in younger patients, with the youngest being an 11-year-old.^30^ The solitary plasmacytomas of the bone (SBP) and extramedullary tissue (SEP) are more common than multiple plasmacytomas. Although, SEP is less common than SBP, it constitutes 4% of all plasmacytomas and arises from the plasma cells of mucosal surfaces, particularly in the upper airway tract. Rare occurrences of ocular plasmacytomas involve the orbit, conjunctiva, and eyelid presenting as proptosis, ptosis, diplopia, and reduced visual acuity.^31–35^ Epiphora resulting from bilateral lacrimal sac involvement by extramedullary plasmacytomas has also been reported. Ocular involvement can either occur as SEP of orbital soft tissue, through orbital invasion of nasopharyngeal SEP, or via local destruction of orbital bone by SBP.^35^

Extramedullary plasmacytomas may also be seen in up to 30% of myeloma patients during their disease course.^36^ In SPs, SBP has a high risk of progression, and most cases have a pre-existing generalized disease.^37^ Radiotherapy with >40 Grays is the treatment of choice, and a high rate of local disease control is observed, with prolonged disease-free survival (30% in SBP and 65% in SEP).^38^ Factors that adversely affect prognosis are age >65, treatment with radiation dose <40 Gy, and bony involvement.^39^ Solitary orbital plasmacytomas also respond well to irradiation but can develop into systemic MM within 2–3 years post-treatment.^40,41^ Some studies have shown that a combination of radiotherapy and surgery has a better overall prognosis than either monotherapy, although results are yet to be replicated in more extensive studies.

### Multiple myeloma

Multiple myeloma (MM) is a malignant plasma cell disorder characterized by ≥10% clonal plasma cells in the bone marrow or biopsy-proven bony or soft tissue plasmacytoma along with either organ impairment (CRAB features) or presence of a biomarker associated with progression to end-organ damage.^42^ It has an age-standardized incidence of around 4 per 100,000, making it the second most common hematological malignancy after lymphoma and accounting for 1% of all cancers.^43,44^ The median age of onset is 66 years, and the most common presenting symptoms of MM include fatigue and bone pain.^45^ The ocular manifestations associated with MM have been extensively reported and are occasionally the symptoms in the early stages of the disease.^46–51^ Moreover, ocular findings can also be the first sign of relapse of previously diagnosed MM after insufficient chemotherapy.^52^

The ophthalmic symptoms occur due to direct deposition of immunoglobulin light chains in ocular tissues (as reported in the cornea, conjunctiva, ciliary pigment epithelium, ciliary body, and subretinal pigment epithelium, or as a manifestation of hyperviscosity syndrome.^53^ Increased circulating protein content and abnormal polymerization of monoclonal immunoglobulins occurring in up to 2–6% of MM patients.^47,54–59^ The eye may also be involved by extramedullary plasmacytomas causing compression of tissues, metastatic infiltration of ocular tissues, or paraneoplastic retinal degeneration.^51,60–62^ The aforementioned pathophysiological mechanisms result in the following clinical manifestations of MM affecting various ocular structures.

Orbital involvement is primarily in the form of plasmacytomas and necrotizing xanthogranulomas (NXG). NXG is a rare histiocytic disease characterized by indurated, non-tender, dermal, and subcutaneous nodules and plaques infiltrating periorbital structures, including eyelids. Although, not completely understood, deposits of protein and immune complexes elicit a granulomatous response, likely leading to NXG formation.^63^ These lesions respond well to systemic corticosteroids, melphalan/cyclophosphamide, and other cytotoxic therapy.^64,65^ Orbital involvement is rare and often the first presenting symptom of myeloma, although it can be a sign of relapsing disease.^66^ It classically presents as unilateral proptosis, although diplopia, decreased vision, extra-ocular movements, orbital swelling, chemosis, pain, and ptosis are also seen. Eyelids can be involved with lesions similar to ecchymosis superficial planar xanthomatosis, and necrobiotic xanthogranulomas.^51,67,68^ A few cases of metastatic myeloma presenting as a necrotizing eyelid lesion has been reported. Conjunctival involvement, although rare, presents as a salmon conjunctival patch having dense stromal infiltration with myeloma cells on histopathology.^68–71^ Conjunctiva can also be affected by calcium deposits due to myeloma-associated hypercalcemia or by sludging of conjunctival vessels by red blood cells due to hyperviscosity syndrome. The myeloma-associated conjunctival salmon patch is susceptible to chemotherapy and autologous stem cell transplantation (ASCT), avoiding the need for surgical excision.^72,73^

Corneal involvement is seen in up to 1% of the population with gammopathies. It encompasses the presence of corneal opacities from light chain deposits, vortex keratopathy, and paraproteinemic keratopathy (throughout the MGUS, SMM, and MM spectrum).^54,58,67,74^ Most corneal immunoglobulin deposits are crystalline but can also be amorphous, hyaline, or granular. The crystal depositions can mimic corneal findings of ocular cystinosis, but irritation is absent in depositions from myelomatous crystals.^58,75,76^ These deposits can involve all layers of the cornea, whereas dystrophies are limited to merely one layer. Patients may be asymptomatic or present with diminished vision, photophobia, and glare. The signs of corneal involvement in MM include corneal epithelial irregularity, stromal opacity, and visualization of deposits under slit-lamp examination.^47,77^

The pathophysiology underlying the immunoglobulins reaching the cornea is not completely understood. A few studies show light chains dissolved in tears being washed over the cornea with subsequent diffusion and deposition, immunoprotein production by keratocytes, and deposition from limbic vasculature.^27,78^ Evaluation and follow-up with corneal confocal microscopy can be used to evaluate, diagnose, and follow up on these changes. The degree and severity of involvement drive the treatment of crystalline keratopathy. In cases with minimal visual symptoms no intervention may be required, whereas severe, irreversible corneal involvement may require penetrating keratoplasty. Recurrence is probable if circulating immunoglobulins persist; therefore, targeting the underlying malignancy is imperative.^79^

Another infrequent phenomenon of dense copper accumulation in the Descemet membrane and the anterior lens capsule is seen with myeloma, presenting with the blurring of vision and subjective change in “iris color”. Slit-lamp examination and confocal microscopic imaging reveal a central, yellow-brown discoloration of each cornea (vs. Wilson's disease, where the discoloration is annular and peripheral). It is also associated with discoloration and thickening of the anterior lens capsule. Further evaluation by checking serum immunoglobulins and treatment should target the underlying myeloma are recommended. Descemet-stripping endothelial keratoplasty or full-thickness keratoplasty and cataract extraction and intraocular lens implantation temporarily restores the ocular symptoms.^21,74,80^ The sclera can be colonized by direct myeloma cell infiltrates in metastatic disease and plasma cell leukemia.^81^ Posterior scleritis is seen with myeloma and presents as progressing, unilateral or bilateral eye pain and redness.^77,81^ Non-invasive testing such as optical coherence tomography and B-scan ultrasonography can help with the diagnosis of posterior scleritis, revealing thickened sclera and a classic “T-sign”, but blood tests for myeloma clinch the diagnosis. The management consists primarily of steroids with a long tapering course. It can be recurrent without treatment of the underlying myeloma, and with anti-myeloma therapy, patients stop experiencing attacks of the same.^69,82^ Apart from these manifestations, NXGs may appear in episcleral tissues and trigger symptoms of recurrent scleritis and episcleritis.^83^

Bronstein and colleagues were the first to discover aggregates of plasma cells in the iris and choroid.^81^ Knapp and colleagues further found bilateral microcysts of the iris pigment epithelium in 6 of the 11 myeloma patients included in their study.^84^ These cysts are often observed without direct malignant ocular involvement, but the electrophoresis of their aspirate resembles the serum electrophoresis of myeloma patients. On physical examination, they are rarely seen and can occasionally rupture to drain contents into the vitreous chamber. In established myeloma cases, infiltration of the iris is observed that stimulate non-granulomatous uveitis which can be confirmed with an anterior chamber paracentesis.^84,85^ The presenting symptoms in these patients include conjunctival hyperemia and painless loss of vision. The symptoms may be improved with systemic chemotherapy and treatment of underlying myeloma.^32^ Recurrent iridocyclitis leading to angle-closure glaucoma has also been reported in a MM patient.^86^

MM masquerading as bilateral macular detachments has been reported, presenting as reduced vision spanning over weeks to months. The diagnosis is made with dilated fundoscopy, optical coherence tomography, and fundus fluorescein angiography, which shows an absence of fluorescein leakage around the FAZ. The pathogenesis is attributed to immunoglobulin extravasation from retinal vessels and subsequent deposition, raising oncotic pressures and leading to serous macular detachments.^26,50,87^ Majority of these patients are diabetics, which is explained by the fact that coexisting diabetes permits a lower threshold for immunoglobulin leakage than in nondiabetics.^60^ The chemotherapy gradually improves the symptoms and restores vision in these patients.^87^ Bilateral pseudovitelliform macular lesions, possibly representing subretinal immunoglobulin deposits, were also reported in one case before myeloma diagnosis.^88^

**Figure 2.**
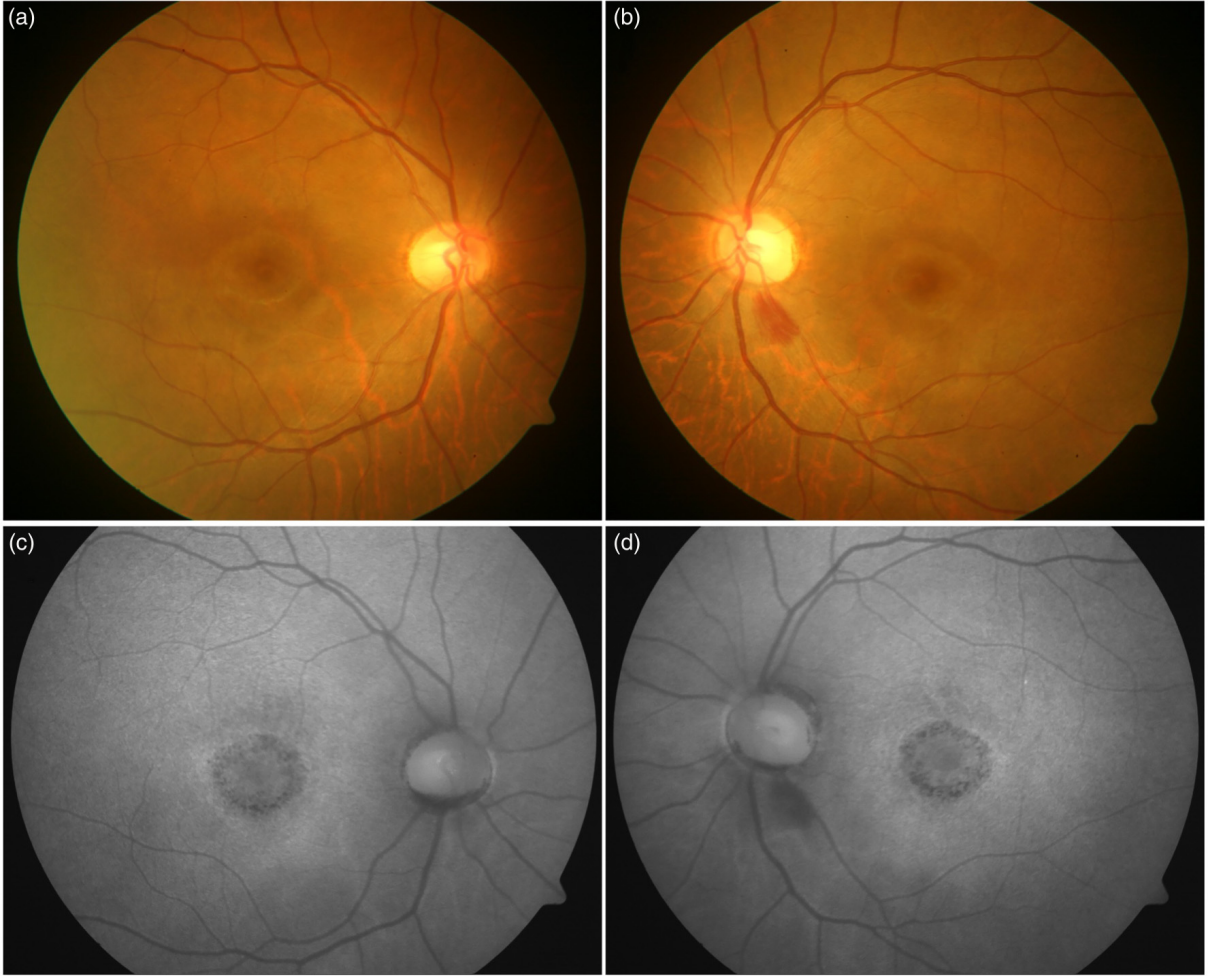
The figure shows a patient with multiple myeloma associated paraproteinemia (66-year-old Asian Indian female). The fundus photograph shows central area of diffuse orange area suggestive of retinal atrophy and visible underlying choroid, surrounded by a ring of lightly pigmented area, followed by a dark ring (in a target pattern) (a and b). The corresponding fundus autofluorescence image (FAF) also shows a ring pattern with a large central atrophy of retinal pigment epithelium (RPE) with hyper-autofluorescent outer ring.

**Figure 3.**
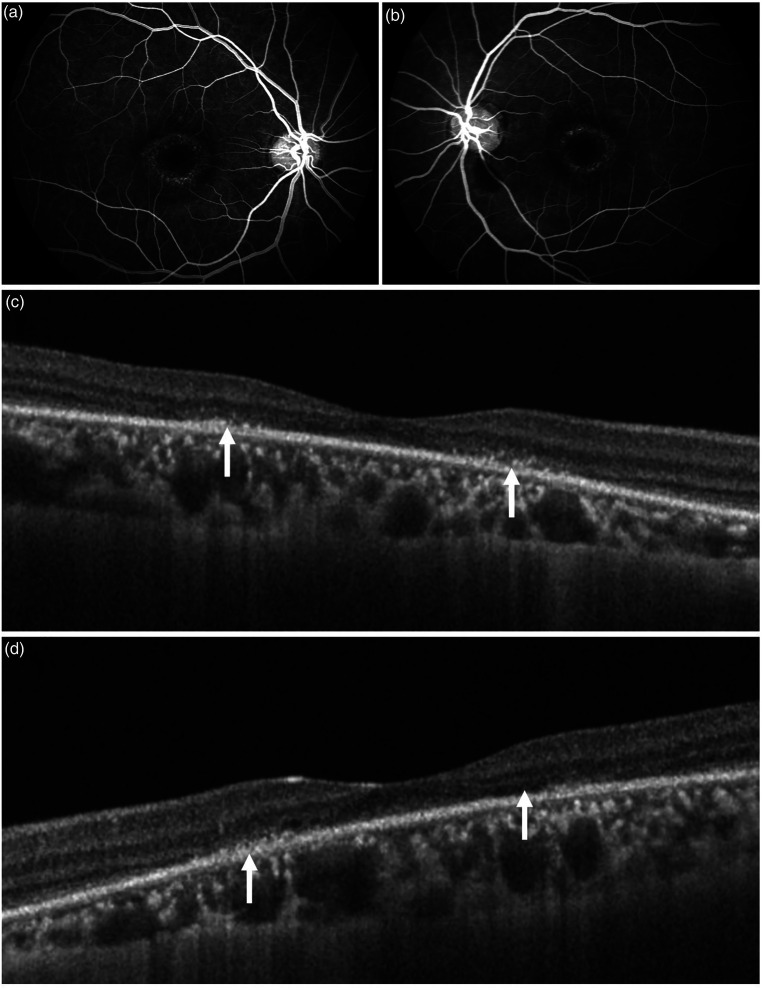
The figure shows the same patient as in figure 2 with positive findings on fluorescein angiography (FA) and optical coherence tomography (OCT). FA shows central hyperfluorescence in the macular region which is symmetrical in both eyes (a and b). On OCT, there is presence of hyper-reflective foci in the outer retina along with disruption of the ellipsoid zone, interdigitation zone, and external limiting membrane. Thinning of the retinal pigment epithelium is suggestive of atrophy (c and d).

**Figure 4.**
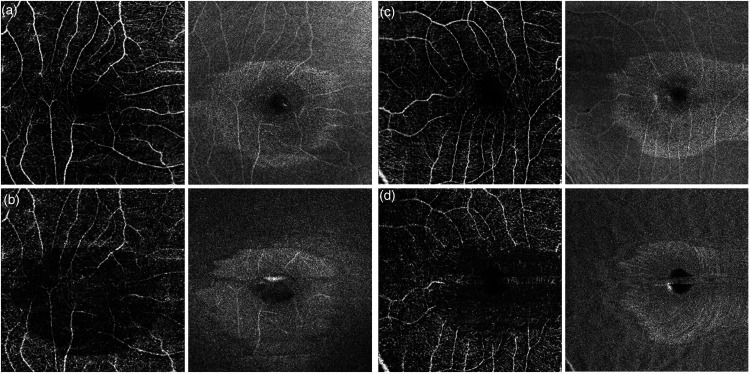
Optical coherence tomography angiography (OCTA) of the same patient as in figure 2. The right eye is represented in panels a and b, and left eye is panels c and d. The superficial retinal plexus shows largely preserved superficial capillary plexus and no evidence of signal loss on corresponding structural *en face* image (a and c). However, the deep retinal vascular plexus shows distinct well-defined loss of deep retinal capillaries (b and d) suggestive of deep retinal ischemia.

Simultaneous bilateral central retinal vein occlusion is seen as the initial presentation of MM and presents with sudden painless loss of vision. It is often associated with hyperviscosity retinopathy, showing features like Roth spots and cotton wool spots, extensive intraretinal hemorrhages, dilated and tortuous central retinal vein, and disc swelling. MM is an important consideration in younger patients with bilateral vein occlusions with blood work signifying paraproteinemia.^89,90^ Paraproteinemia can be associated with deep retinal ischemia visible on multimodal imaging (Figures 2–4). Hyperviscosity may also present as non-ischemic retinal vein occlusion with subsequent retinal artery occlusion.^13,85^

Optic nerve may also be involved in myeloma via direct nerve infiltration with myelomatous cells, presenting as a painless vision reduction. Disc swelling can be visualized on fundoscopy, and vision recovery is rapid with the initiation of chemotherapy.^91^ It may also be the first presenting sign of recurrence while being on maintenance chemotherapy.^92^

Diplopia and visual disturbances are present after the involvement of cranial nerves II, III, and VI and the extraocular muscles. The sixth cranial nerve is found to be most commonly involved.^93,94^ Temporal arteritis and retinal vasculitis have been reported in association with MM cause blurry vision and reduced visual acuity.^68^ Early clinical suspicion of plasma cell dyscrasias in otherwise unexplained ocular symptoms can lead to a quicker diagnosis. The ophthalmic evaluation at the time of diagnosis of MM can help prevent associated ocular morbidity.

### Waldenström macroglobulinemia

Waldenström macroglobulinemia (WM) is a malignant B-cell neoplasm caused by overproduction of monoclonal IgM and lymphoplasmacytic infiltration of the bone marrow.^95^ WM has an incidence of 3 per million persons per year and accounts for 1–2% of all hematologic cancers.^96^ Ocular manifestations in WM were first reported by Waldenström himself in 1952.^97^ These occur due to the circulating IgM leading to hyperviscosity of the plasma.^85,98^ The patients suffering from hyperviscosity ocular symptoms typically present with unilateral or bilateral reduced visual acuity and are reported to have dilatated retinal vessels, intraretinal hemorrhages, retinal vein occlusions, and optic disc edema.^98–101^ Maculopathy with treatment-resistant macular detachment and edema have also been reported in WM patients.^102^ There is a linear relationship between the viscosity of blood with the severity of ocular signs.^99,102,103^ Lower serum viscosities are associated with early hyperviscosity retinopathy, like hemorrhages in the far peripheral retina and retinal venous dilatation. Thus, indirect ophthalmoscopy with scleral depression and retinal vessel diameter measurement is important in diagnosed patients of WM to detect the early signs of hyperviscosity.^99^

The treatment of WM includes plasmapheresis and systemic chemotherapy, which are associated with improvement in symptoms of hyperviscosity retinopathy.^99,104^ However, WM-associated maculopathy persists despite systemic chemotherapy, immunotherapy, and intravitreal bevacizumab injections.^102^

### Systemic amyloidosis

Amyloidosis is a group of protein misfolding disorders where faulty protein deposits as insoluble fibrils in the extracellular tissues, causing various clinical manifestations.^105^ The location of the affected tissues determines whether it is systemic or localized. Systemic amyloidosis is further classified based on the biochemistry of the precursor protein into amyloid light chains (AL), also historically known as primary amyloidosis, serum amyloid A (AA), also traditionally known as secondary amyloidosis, familial transthyretin amyloidosis (ATTR), and hemodialysis related amyloidosis (Aβ2M).^106–108^

The monoclonal proliferation of plasma cells results in the deposition of the λ and κ light chains of immunoglobulin. In the eye, primary amyloidosis has been known to involve extrinsic ocular muscles, eyelids, conjunctiva, temporal artery, retina, vitreous, orbital fat, choroid, trabecular meshwork, and cranial nerves.^109–119^ The ocular manifestations range from glaucoma, eye pain, decreased vision, ptosis, binocular diplopia, floaters, swollen eyelids, and conjunctival masses, ophthalmoplegia, drusenoid deposits, serous retinal detachment, sub-retinal fluid deposition, bilateral lacrimal deficiency, and occlusion of choriocapillaris.^120–122^ The symptoms are explained by four mechanisms: neural protein deposits leading to neuropathy, vessels protein deposits leading to obstruction and vascular fragility, the mass effect of amyloid leading to structural distortion, and vitreous opacification.^120^ A study found amyloid as a mass in the plica semilunaris, and on subsequent evaluation, the patient developed systemic symptoms.^112^ As seen in other plasma dyscrasias, this case report emphasized the importance of ocular manifestations as the first sign of systemic amyloidosis. An earlier study by the National Institutes of Health also corroborated ophthalmic symptomology to be an earlier sign of the systemic disease process.^111^ The adnexal involvement in these patients is seen as waxy eyelid papules with purpura, proptosis, diplopia, ptosis, accommodative paresis, keratoconjunctivitis sicca, and upper lid masses.^123^ In these patients, scalloped pupils or amyloid particles in the pupillary margin may also be observed. Additionally, amyloid deposition in trabecular meshwork leads to increased intraocular pressure. In the cornea, two different studies found that systemic amyloidosis presents as lattice stromal dystrophy (Type-2 Meretoja syndrome).^124,125^ AA amyloidosis usually occurs due to an inflammatory or infectious process.^106–108^ It has been shown to have little or no ophthalmic involvement.^111,119,126^ ATTR amyloidosis has a similar clinical picture to AL amyloidosis with peripheral neuropathy, cardiomyopathy, carpal tunnel syndrome, and autonomic neuropathy.^127^ In contrast to AA amyloidosis, transthyretin amyloidosis is more commonly associated with ocular damage. Reynolds and colleagues found that 46% of women displayed ocular symptoms compared to only 15% of male patients.^115^ The ocular manifestations included decreased visual acuity, vitreous amyloid, glaucoma, neurotrophic keratitis, and tortuous retinal vessels. The study found that vitrectomy significantly helped with the improvement of visual acuity. The recent advances in proteomics allow for reliable typing of amyloid precursor protein with high specificity and sensitivity.^128^ Local surgical and pharmacological interventions, like vitrectomy alone, are insufficient for a patient with systemic amyloidosis and depends on systemic therapy consisting of IV, oral, or topical steroids.^23,129^

### Polyneuropathy, organomegaly, endocrinopathy, M protein, and skin abnormalities (POEMS) syndrome

Polyneuropathy, organomegaly, endocrinopathy, M protein, and skin changes (POEMS) syndrome is a rarer subset of plasma cell dyscrasias.^130^ Kaushik *et al*. assessed 33 patients with ophthalmic signs and symptoms of POEMS at a tertiary care center and observed that 29% of asymptomatic patients had bilateral optic disc edema (ODE) and presented elevated VEGF levels and lumbar puncture opening pressure.^131^ A recent prospective study from China confirmed Kaushik and colleagues’ findings showing high incidence of papilledema among 41 patients diagnosed with POEMS.^132^ The same study also showed elevated VEGF levels. These studies reported retinal damage consisting of hemorrhages, cotton wool spots, retinal detachment, and macular edema. Zhang and colleagues showed a positive correlation between VEGF levels, retinal nerve fiber layer thickness, and peripapillary retinal thickness. All ocular symptoms showed significant improvement with dexamethasone and lenalidomide therapy. Another study including 94 patients with POEMS showed papilledema as an early presenting symptom and identified a strong correlation between increased intracranial pressure and elevated CSF protein with papilledema.^133^ In this study, papilledema and monotherapy with oral corticosteroids were associated with poor prognosis.

Additional case reports have been published to substantiate the relationship between papilledema as an initial presenting symptom and POEMS Syndrome for patients between 16 and 62 years.^134–140^ The same studies have also outlined the presence of cystic macular edema (CME).^138–141^ The etiology of ODE is thought to be elevated intracranial pressure and CSF protein, increased vascular permeability due to cytokines, inflammation (vasculitis), and infiltration.^130,136^

Decreased visual acuity is another common presentation in conjunction with ODE, blurred vision, enlarging blind spot, retinal detachment, floaters, disc hyperfluorescence, altered visual evoked potential, photopsia, central retinal artery occlusion, chalky white optic disc, and choroidal filling defects.^142–146^ In a prior case report, Barry and colleagues detected a progressively enlarging blind spot in addition to ODE, which vastly improved with oral steroid therapy.^142^ They also observed the previously mentioned elevated protein and pressure findings in CSF. On optical coherence tomography, the findings of papilledema of all retinal layers, bilateral peripapillary serous retinal macular detachment, and altered thickness of the peripapillary retina and nerve fiber layer were seen.^147^ Besides systemic therapy, very few local ocular-specific treatments have been ascertained. Vitrectomy and vitreous triamcinolone acetonide injection cmay be considered to reduce retinal edema and prevent serous detachment.^139^

### Cryoglobulinemia

Cryoglobulinemia refers to immunoglobulins that precipitate in the serum at temperatures < 37 °C.^148^ Brouet and colleagues classified cryoglobulinemia into three categories. Type I involves a single type of monoclonal immunoglobulin, either IgM or IgG. Type II and Type III are categorized as mixed cryoglobulinemia because Type II consists of polyclonal IgG and monoclonal rheumatoid factor (IgM), and Type III consists of polyclonal IgG and polyclonal rheumatoid factor (IgM).^149^

The mechanism of cryoglobulin damage occurs either by depositing cryoglobulins in microcirculation or immune-mediated pathways.^148^ The symptoms range from the classic triad of purpura, arthralgia, weakness associated with cryoglobulinemic vasculitis, skin ulcers, Raynaud's phenomenon, arthritis, sicca syndrome, and peripheral neuropathy.^148,150^ In addition, renal involvement is seen in about 15–30% of the cases, and gastrointestinal, and CNS involvement remains exceedingly rare.^151^ The first cases of cryoglobulinemia with ocular symptoms were first described in 1950; however, a quantitative assessment of the frequency of ocular symptoms associated with cryoglobulinemia has never been performed. The ophthalmic manifestations in cryoglobulinemia are mainly caused by hyperviscosity syndrome and manifest as retinal hemorrhages and cotton wool spots, leading to blurry vision and eventual visual loss.^59,152^ The primary treatment consists of plasma exchange regardless of the cryoglobulinemia type.^152^ Typically, patients present with unilateral visual loss due to retinal vein occlusion as the initial manifestation. The patients are treated with monthly intravitreal ranibizumab and oral prednisone, resulting in undetectable serum cryoglobulins and improved visual acuity. Sebrow and colleagues reported an unusual case of perivenous retinal whitening and retinociliary venous sparing, secondary to central retinal vein occlusion, has also been reported, with underlying concomitant antiphospholipid syndrome and type 2 cryoglobulinemia.^153^ Central retinal artery occlusion has also been reported secondary to cryoglobulinemias, and treatment with anticoagulation and prednisone led to complete restoration of visual acuity.^154^ In some cases, anterior segment involvement may also be seen. Telander and colleagues reported two patients with anterior segment ischemia secondary to underlying cryoglobulinemias. The patients presented with iris neovascularization, or rubeosis, in the absence of retinal ischemia, and one of the two patients experienced resolution with treatment of underlying lymphoma.^155^ In a few patients, anterior uveitis has also been observed, and it improves after a standard treatment regimen consisting of plasmapheresis and rituximab.^156^ Purtscher's retinopathy without trauma was reported in two males with chronic hepatitis C and cryoglobulinemia.^157,158^ In both cases, all retinal changes, except optic atrophy, resolved in months, and partial visual acuity was restored with treatment. The underlying pathogenesis is due to deposition of cryoglobulins in small vessels, including retinal vessels causing severe local inflammation. In these cases, intravascular clumping of leukocytes resulted in microthrombi formation in response to abnormal activation of complement. Necrotizing scleritis with peripheral ulcerative keratitis has been associated with mixed cryoglobulinemia in a few cases. These patients had underlying Hepatitis C and responded to high-dose systemic corticosteroids.^159–161^ One case with underlying MGUS has been reported in association with retinal vasculitis, and the resolution of symptoms with high doses of topical dexamethasone and artificial tears was reported.^162^ Corneal involvement has also been reported with subepithelial cryoglobulin deposits, presenting symptoms like blurring vision, gradual decline in vision, photophobia, and progressive hyperopia.^161,163–166^ In one case, debridement of deposits with phototherapeutic keratectomy led to significant improvement in visual acuity. Kremer and colleagues hypothesized that precipitates in the subepithelial cornea could be attributed to this region being one of the coldest areas in the body.^165^

## Conclusion

This review outlines the ocular manifestations of plasma cell dyscrasias, namely the pre-malignant conditions: smoldering MM and MGUS, plasmacytomas, multiple myeloma, Waldenström's macroglobulinemia, systemic amyloidosis, POEMS syndrome, and cryoglobulinemia. The literature underlines the importance of detecting ocular signs in the swift diagnosis of plasma cell dyscrasias and preventing irreversible vision. However, the ocular involvement in a patient with plasma cell dyscrasias is wide-ranging; therefore, high clinical suspicion is essential for accurate diagnosis, specifically in asymptomatic patients. Moreover, some ocular symptoms have been reported more frequently than others, and it is vital to analyze the full spectrum of symptoms to assess the patient's clinical outcomes accurately. While systemic findings associated with plasma cell dyscrasias have been extensively described, the ocular complications have not been thoroughly investigated. Therefore, retrospective and prospective studies with large cohorts are necessary to clearly outline the ocular symptoms and understand the underlying pathological processes in these patients.
